# Tristetraprolin, a Potential Safeguard Against Carcinoma: Role in the Tumor Microenvironment

**DOI:** 10.3389/fonc.2021.632189

**Published:** 2021-05-07

**Authors:** Diwen Zhang, Zhigang Zhou, Ruixia Yang, Sujun Zhang, Bin Zhang, Yanxuan Tan, Lingyao Chen, Tao Li, Jian Tu

**Affiliations:** ^1^ Institute of Pharmacy and Pharmacology, University of South China, Hengyang, China; ^2^ Department of Biomedical Sciences, City University of Hong Kong, Hong Kong, China; ^3^ The Second Affiliated Hospital of Guilin Medical University, Guilin, China; ^4^ Department of Experimental Animals, University of South China, Hengyang, China; ^5^ Pharmacy School of Guilin Medical University, Guilin, China; ^6^ Shanghai Veterinary Research Institute, Chinese Academy of Agriculture Science, Shanghai, China

**Keywords:** tristetraprolin (TTP), RNA binding protein, potential safeguard, carcinoma, tumor microenvironment (TME)

## Abstract

Tristetraprolin (TTP), a well-known RNA-binding protein, primarily affects the expression of inflammation-related proteins by binding to the targeted AU-rich element in the 3’ untranslated region after transcription and subsequently mediates messenger RNA decay. Recent studies have focused on the role of TTP in tumors and their related microenvironments, most of which have referred to TTP as a potential tumor suppressor involved in regulating cell proliferation, apoptosis, and metastasis of various cancers, as well as tumor immunity, inflammation, and metabolism of the microenvironment. Elevated TTP expression levels could aid the diagnosis and treatment of different cancers, improving the prognosis of patients. The aim of this review is to describe the role of TTP as a potential safeguard against carcinoma.

## Introduction

Tristetraprolin (TTP), also known as ZFP36, NUP475, G0/G1 switch regulatory protein 24 (GOS24), and TPA-inducible sequence 11 (TIS11), is a well-known RNA-binding protein (1, 2). It is an early immediate response gene located on chromosome 19q13.2, contains one intron and two exons and encodes a 1.7 kb messenger RNA (mRNA) transcript and a 34 kD protein (3). The unique features of the structure of TTP include three proline-rich motifs, two conserved tandem zinc-finger (TZF) domains with the CX8CX5CX3H sequence, and several serine/threonine phosphorylation sites such as S66, S88, T92, S169, S186, S197, and S228 (4, 5).

TTP acts at the transcription, translation, and post-transcription cellular levels (6). Post-transcription regulation, during which TTP targets the AU-rich element (ARE)-containing mRNA and affects its stability, is essential for cells to rapidly respond to intracellular and extracellular stimuli (7). TTP optimally combines with 9-mer UUAUUUAUU and the relevant binding sequence at the 3′-UTR of the target mRNA through the TZF domain. Thus, TTP promotes the instability and degradation of mRNA (8). Additionally, TTP can shorten the poly (A) tail of the target mRNA of cytokines and mediate mRNA decay through the 3-5 exosome and 5-3 Xrn1 exonuclease pathways (9). In the 3-5 pathway, TTP identifies and recruits the exosome to ARE-mRNAs (10). After the 7-methyl guanosine cap is removed by TTP in the decapping complex, decay progresses in the 5-3 pathway. Subsequently, ARE transcripts are transferred to the processing body by TTP (11). Therefore, when factors related to inflammation and cancer are overexpressed, TTP interacts with specific mRNA to repress several crucial gene transcripts and rapidly degrade ARE-directed binding proteins (12).

In recent years, TTP has been found to be associated with some pro-inflammatory and cancer-promoting proteins, such as tumor necrosis factor (TNF) alpha, C-X-C motif chemokine receptor 4, mRNA-decapping enzyme 2, and enhancer of mRNA-decapping protein 3 (13, 14). By interacting with specific mRNA, TTP blocks post-transcriptional activity and protein synthesis and downregulates the expression of these mRNAs to counteract the pro-tumorigenic effects of these tumor growth factors (15, 16).

## Roles of TTP in Carcinoma

**Figure 1:**
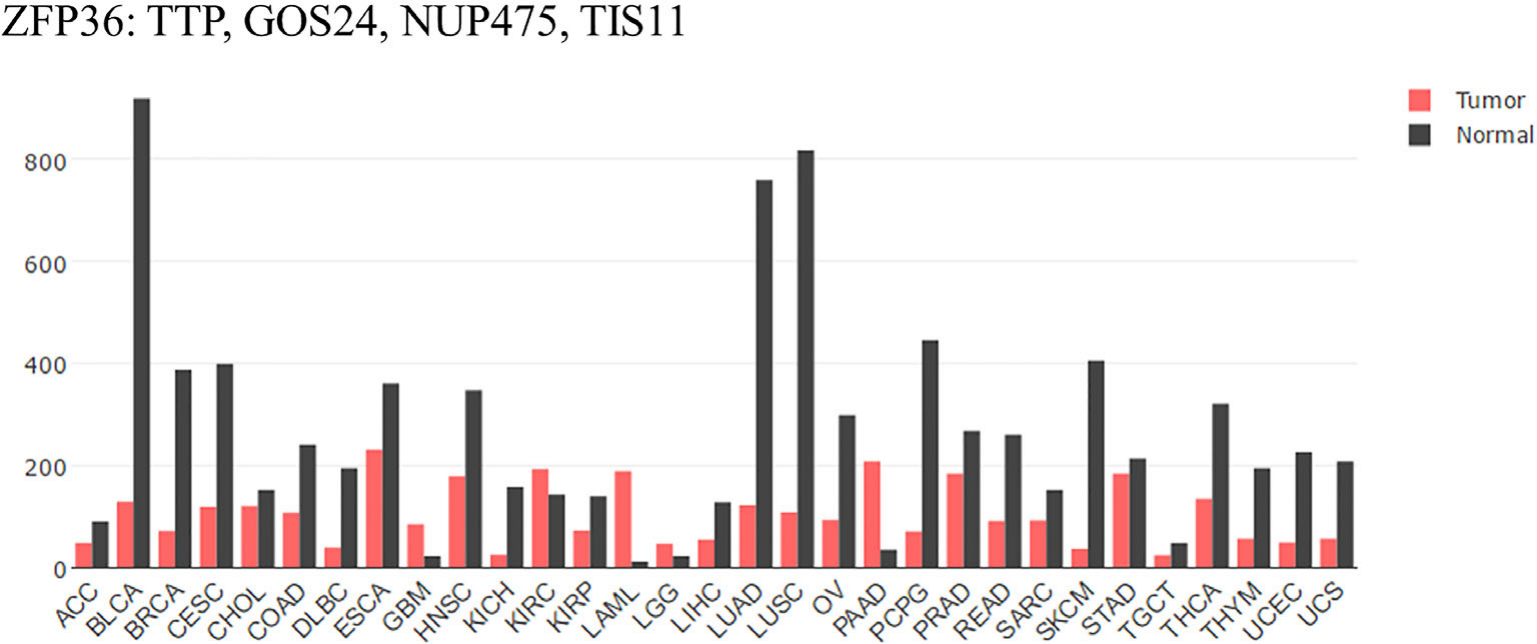
TTP/ZFP36 expression in cancer and normal tissues analyzed by GEPIA. Compared with normal tissues analyzed by GEPIA; TTP mRNA expression is significantly lower in the tissues of most forms of carcinoma. ACC, Adrenocortical carcinoma; BLCA, Bladder urothelial carcinoma; BRCA, Breast invasive carcinoma; CESC, Cervical squamous cell carcinoma; CHOL, Cholangiocarcinoma; COAD, Colon adenocarcinoma; DLBC, Lymphoid Neoplasm Diffuse Large B-cell Lymphoma; ESCA, Esophageal carcinoma; GBM, Glioblastoma multiforme; HNSC, Head and Neck squamous cell carcinoma. KICH, Kidney Chromophobe; KIRC, Kidney renal clear cell carcinoma; KIRP, Kidney renal papillary cell carcinoma; LAML, Acute Myeloid Leukemia; LGG, Brain Lower Grade Glioma; LIHC; liver hepatocellular carcinoma; LUAD; lung adenocarcinoma; LUSC; lung squamous cell carcinoma; OV, ovarian serous cystadenocarcinoma; PAAD, pancreatic adenocarcinoma; PCPG, Pheochromocytoma and Paraganglioma; PRAD, Prostate adenocarcinoma; READ, Rectum adenocarcinoma; SARC, Sarcoma; SKCM, Skin Cutaneous Melanoma; STAD, Stomach adenocarcinoma; TGCT, Testicular Germ Cell Tumors; THCA, Thyroid carcinoma; THYM, Thymoma; UCEC, Uterine Corpus Endometrial Carcinoma; UCS, Uterine Carcinosarcoma (17).

Compared with that of normal tissues analyzed by GEPIA (**Figure 1**), mRNA expression of TTP is significantly lower in adrenocortical carcinoma (ACC), bladder urothelial carcinoma (BLCA), breast invasive carcinoma (BRCA), cervical squamous cell carcinoma (CESC), and colon adenocarcinoma (COAD) tissues. Furthermore, the expression level is always associated with clinicopathological features, overall survival rate, and patient prognosis (18–22). Some reports have illustrated that Area Under Curve (AUC) of TTP in prostate cancer is 0.84 to 0.85 (The closer to 1.0, the higher the authenticity of the detection is) using bioinformatics data combined with clinical model. Besides, lower level of TTP is related to more aggressive phenotype in HCC. Loss of TTP will show high correlation with late-stage, high-grade of HCC (23, 24). Therefore, TTP could be considered a tumor suppressor in most cancers, and upregulating TTP expression might help improve prognosis. Besides, TTP is involved in many cancer processes, including the proliferation, apoptosis, and metastasis. In the following, a detailed role of TTP will be stated respectively.

### TTP Inhibits the Proliferation

The abnormality of protooncogene and tumor suppressors results in the tumorigenesis. Next, the uncontrolled cell replication and division stimulated by cancerous signal transduction promotes the continual proliferation. In this part, TTP has been reported from two sides, one is as a post-transcriptional regulator in plasma, and the other is as a transcriptional regulator in the nucleus (25).

#### TTP as a Post-Transcriptional Regulator in Plasma

TTP can inhibit the progression of several cancers (26–28). In 2009, TTP was first described to impair nuclear translocation of nuclear factor kappa-B (NF-κB) p65 (29). Thus, TTP and NF-κB target genes are also involved in mediating inflammatory responses in the cell cycle (30). In 2015, TTP was found to affect the c-Jun/Wee1 axis at the transcriptional level in an NF-κB-dependent manner in breast cancer. Being a transcriptional co-repressor that binds with the C-terminal Zn finger of NF-κB p65, TTP blocks p65 translocation. As a result, the decreased c-Jun level and increased Wee1 expression could delay the transition from S to G2 in the cell cycle, thereby reducing cell proliferation (26). Three years later, the same research group found that TTP inhibited the proliferation of lung adenocarcinoma cells by inducing cell cycle arrest in the S phase, which might be related to autophagy. Soon after, TTP overexpression was shown to downregulate autophagy-related mRNA and protein levels, such as those of Beclin1 and LC3II/I in lung cancer cells (27). In addition, TTP could also inhibit the proliferation of non-small cell lung cancer. Being an inhibitor of histone deacetylases (HDAC), sodium butyrate (NaBu) could downregulate Cyclin B1 expression by accelerating TTP binding with the 3′-UTR of Cyclin B1, which prevents the cell cycle transition from G1 to S and G2 to M (28).

**Figure 2:**
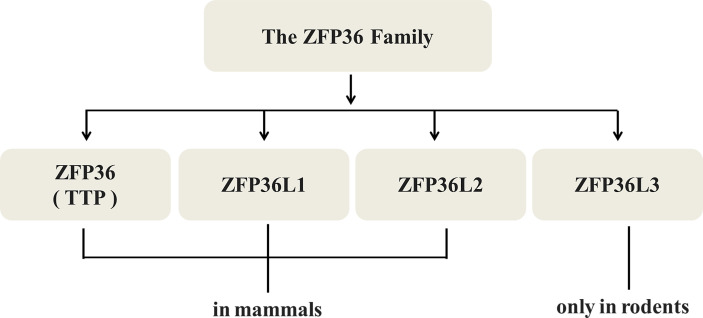
Types of TTP/ZFP36. TTP/ZFP36 along with ZFP36L1 and L2, all members of the ZFP36 family share similar structures and functions in mammals, while L3 is found only in rodents.

Not merely TTP/ZFP36 itself, the other members of ZFP36 family including ZFP36L1 and L2 in mammals, L3 only in rodents (**Figure 2**) are all relevant with proliferation suppression of malignant neoplasms. In colorectal cancer (31), ZFP36L1 and L2 could inhibit proliferation by inducing cell cycle arrest in the G1 phase *via* Cyclin D- and p53-dependent pathways. Forced expression of ZFP36L1 or L2 downregulates cell cycle-related proteins such as Cyclin A, B, and D and p21, but upregulates p53 mRNA. Defects in ZFP36L1 and L2 in TZF did not lead to the inhibition of colorectal cancer proliferation, suggesting that TZF is a key factor in the regulation of ZFP36L1 and L2 (31).

#### TTP as a Transcriptional Regulator in the Nucleus

As a vital transcription factor, TTP transcription also plays a crucial role in inhibiting cell proliferation through mRNA decay. In A549 lung cancer cells, Smad3, a regulatory protein involved in transforming growth factor beta (TGF-β) signaling, can bind to the promoter region of TTP (32). Subsequently, TTP transcription and increased expression leads to a decrease in the mRNA expression of cyclooxygenase-2 (COX-2), a pro-inflammatory factor that can inhibit the proliferation of cancer cells (33). In colorectal cancer cells, HDAC has a negative effect on transcription and silences tumor suppressor genes, such as TTP, in an epigenetic manner. Therefore, HDAC inhibitors such as trichostatin A, SAHA, and NaBu can induce TTP transcription by activating early growth reactive protein 1, another transcription factor (34). Increase in TTP expression was shown to promote COX-2 mRNA degradation and inhibit colorectal cancer cell proliferation (35). In breast cancer cells, TTP expression can be induced by p53 and inhibited by c-Myc (36). In contrast, metformin is believed to promote p53 and suppress c-Myc expression through the AMPK pathway. Moreover, metformin exerts its effects by inducing the promoter activity of TTP transcription (37).

### TTP Induces the Apoptosis

**Figure 3:**
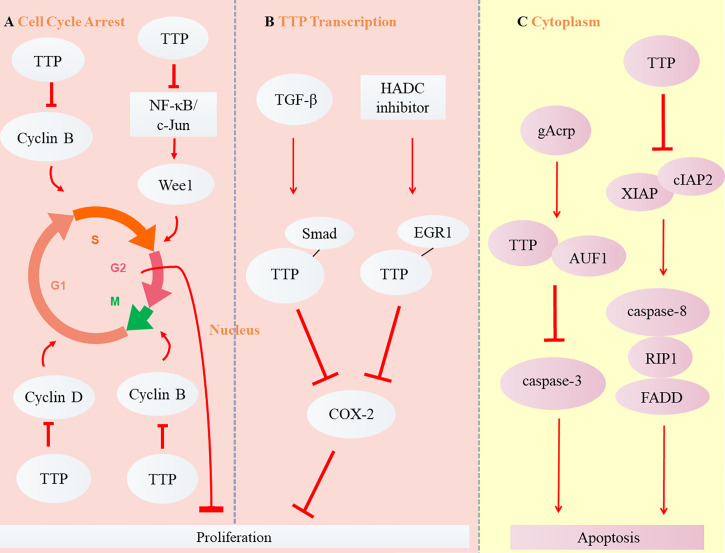
Role of TTP in carcinoma. **(A)** TTP inhibits Cyclin B expression by inducing the arrest of cell cycle from Phase G1 to S or G2 to M, Cyclin D expression in Phase G1, and Wee1 expression from Phase S to G2, thus suppressing the proliferation of cancer cells. **(B)** The transcription of TTP, induced by TGF-β and HADC inhibitor, binds with the other transcription factors, Smad and EGR1. Subsequently, TTP inhibits COX-2 expression and suppresses the proliferation of cancer cells. **(C)** Two cytoplasmic signaling pathways of TTP are involved in the apoptosis of tumor cells.

Different from necrosis, the apoptosis refers to the natural programed cell death. For mammals, it is a critical adjustment to maintain homeostasis by eliminating senile or useless cells (25). B-cell lymphoma 2 (Bcl-2) is an anti-apoptotic protein overabundant in cancer cells that induces tumor resistance to anticancer drugs such as cisplatin (38). TTP enhances cisplatin sensitivity of squamous cell carcinoma of the head and neck (SCCHN) by inhibiting Bcl-2 expression at the post-transcriptional level, thereby enhancing cell apoptosis (39). Globular adiponectin (gAcrp), known for its anti-tumor effects, may accelerate AU-rich binding with the proteins TTP and AUF1. TTP subsequently promotes apoptosis of hepatocellular carcinoma (HCC) by promoting Bcl-2 mRNA instability associated with caspase-3 enhancement (40). Ripoptosome, composed of receptor-interacting protein kinase 1 (RIP1), caspase-8, and Fas-associating protein with a novel death domain (FADD), has been described in recent years as a cell death-inducing complex that aggregates in cancer cells (41–43). Its stability is regulated by the inhibitor of apoptosis proteins (IAPs) E3 ubiquitin ligase, XIAP, cIAP1, and baculoviral IAP repeat containing 3 (cIAP2) (44). TTP reduces the mRNA stability and expression of cIAP2 and XIAP (45, 46), which activates the death complex. RIP1 stimulation promotes the aggregation of RIP1, caspase 8, and FADD, as well as the apoptosis of glioma cancer stem cells (47). However, TTP is not expressed when the promoter is methylated or phosphorylated (48). MAPKAP kinase 2 (MK2) promotes tumor development through TTP phosphorylation (49). The combination of MK2 with a DNA methylation inhibitor (5-aza-dC) or with interferon α restores TTP expression and stimulates the apoptosis of HCC cells by upregulating apoptotic genes such as caspase-4 and -8 (50). The balance between the proliferation and apoptosis of cancer cells is critical for tumor development (51). The TTP signaling pathways in cancers are shown in **Figure 3**.

### TTP Reins in the Metastasis

Metastasis is the main cause of death in cancer patients. When cancer cells exacerbate, they gradually invade in the epithelial cells through extracellular matrix (ECM). Along with the angiogenesis in the microenvironment, the cells spread to distant tissues of body through blood or lymph vessels. Plasminogen activator (uPA) and its receptor uPAR will be the key factors in the invasion. At the same time, TTP can delay the invasion and migration of cancer cells (25). TTP can counteract the invasion of glioma cells by directly binding the 3-UTR mRNA of urokinase uPA and uPAR, thus reducing mRNA abundance and subsequent protein expression (52–54). IL-13 is involved in regulating the proliferation, invasion, and metastasis of cancer cells by activating the PI3K/Akt/mTOR pathway (55, 56). TTP may inhibit the role of PI3K/Akt/mTOR by targeting IL-13 mRNA (20), thereby reducing the growth and invasion of glioma cells. Increased IL-33 expression is closely related to the growth and metastasis of cancer cells (57, 58). In gastric cancer cells, upregulation of TTP inhibits the proliferation, invasion, and migration of cancer cells by negatively correlating with IL-33 expression (22). PIM2, a kinase that is overabundant in cancer, phosphorylates a series of proteins critical for tumor progression (59). By binding to the TZF region of TTP, PIM2 not only promotes the degradation of TTP protein through the ubiquitin-proteasome pathway, but also inhibits the proliferation and migration of breast cancer cells (60).

The delay of metastasis depends on its regulation as well as that of the TTP-HuR axis. At present, thousands of overlapping binding sites between TTP and human antigen R (HuR), which are both antagonistic RNA-binding proteins, have been detected (61). TTP attenuates the targeted mRNAs, while HuR usually plays an essential role in stabilizing the targeted transcripts and promoting mRNA translation (62). Dysregulation of the TTP-HuR axis may increase the factors associated with cancer development (63). Elevation of high mobility group box 1 (HMGB1), a typical damage-related molecule, is often associated with gastric cancer (64). HuR, which has been found to be increased in gastric cancer, promotes HMGB1 expression at the translational level (65). TTP then attenuates the invasion and migration of gastric cancer cells by downregulating HuR expression (63, 65). In non-small cell lung cancer tissues, TTP expression is positively correlated with miR-133b, while HuR expression is negatively correlated with the expression of miR-133b and TTP (66). Consequently, by interacting with TTP-HuR axis (regaining TTP expression but reducing HuR level), miR-133b will rein in the development and progression of cancer (66).

## TTP Acts as a Potential Safeguard Against Carcinoma Due to Its Role in the Tumor Microenvironment (TME)

**Figure 4:**
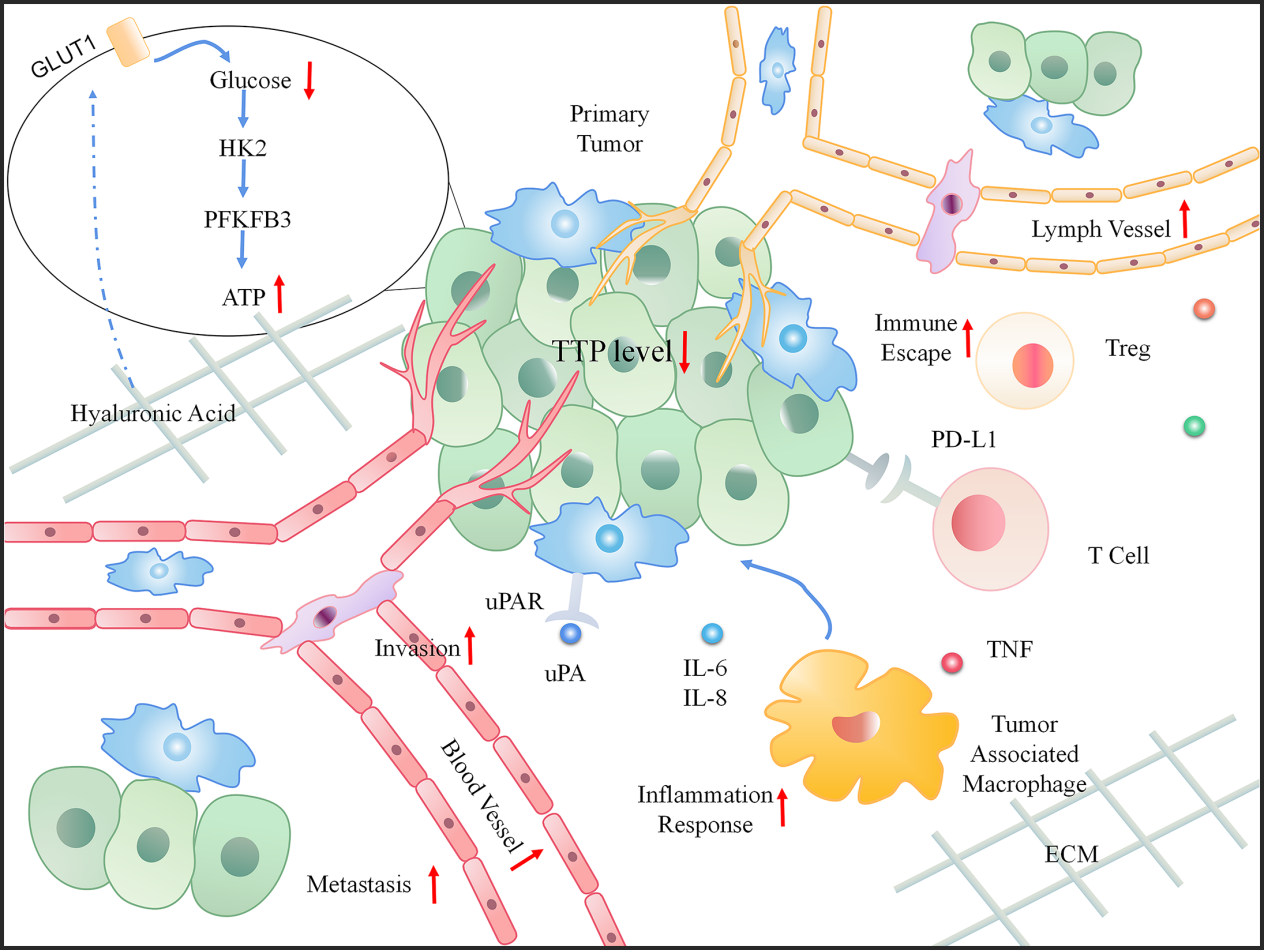
The dynamic change in the tumor microenvironment (TME) when TTP is downregulated. When TTP expression decreases in tumor cells, the primary tumor invades the basement membrane. Moreover, an increased number of blood and lymph vessels aids tumor metastasis. In the microenvironment, the increasing inflammatory response, immune escape, and energy also provide a favorable environment for tumor progression.

Tumor development depends not only on genetic changes, but also on changes in the constituents of the TME, such as cytokines, growth factors, hormones, extracellular matrix (ECM), blood vessels, and invasive inflammation (67). In particular, the TME can release extracellular signals, promote tumor progression (especially for angiogenesis), and induce peripheral immune tolerance. Inflammation and immunity are the core components that drive the onset and development of cancer (68). Studies have recognized the role of TTP in the TME based on its participation in tumor-related inflammation and immunity, thus affecting the progress of cancer and predicting poor survival (69). The dynamic changes that occur when TTP is downregulated in the TME are shown in **Figure 4**.

### TTP Inhibits Angiogenesis and Lymphangiogenesis

Angiogenesis is the sprouting of new vessels from existing ones during embryogenesis. In the adult, angiogenesis is usually turned on, but only transiently. In contrast, during tumor progression, an ‘angiogenic switch’ is almost always activated and remains on, causing normally quiescent vasculature to continually sprout new vessels, which help sustain expanding neoplastic growths (25). TTP suppresses the metastasis through angiogenesis. TTP has been shown to inhibit the expression of vascular endothelial growth factor (VEGF) and COX-2, which are both key factors that influence angiogenesis in human colorectal cancer (34). Furthermore, galanin receptor type 2 (GALR2) and G-protein-coupled receptor (GPCR) induce the aggressive growth of SCCHN and stimulate angiogenesis through p38 MAPK-mediated phosphorylation of TTP. This allows for a decrease in TTP expression and an increase in the secretion of pro-angiogenic cytokines such as VEGF and IL-6 (70). In addition, resveratrol, a polyphenolic compound that naturally occurs in grapes, peanuts, and berries, induces the mRNA-decaying activity of TTP and suppresses the proliferation, invasion and migration of HCT116 and SNU81 colon cancer cells by activating TTP (45).

Similar to angiogenesis, lymphangiogenesis is the proliferation of a network of lymphatic vessels. As mentioned previously, GALR2 overexpression in SCCHN cells could suppress TTP phosphorylation and increase the secretion of IL-6 and VEGF, thereby promoting angiogenesis (34). However, antiangiogenic effects may be undermined by lymphatic vessel formation (71). The lymphatic system is one of the main routes of distant metastasis and growth of cancer cells (72), among which vascular endothelial growth factor C (VEGF-C) is currently regarded as the most representative lymphangiogenic factor (73). Simultaneously, sunitinib inactivates TTP, prolongs the half-life of VEGF-C, and hastens the progression of lymphatic vessels in clear cell renal cell carcinoma (74).

### TTP Overexpression Prevents the Inflammation in Cancer

Inflammation, a key characteristic of the TME, can play a crucial role in the stages of tumor development, such as initiation, promotion, and metastasis (75). Elevated levels of pro-inflammatory genes, associated with continuous inflammation and tumorigenesis, advance the proliferation, angiogenesis, metastasis, survival, and drug resistance of cancer cells (76, 77). The secretion of IL-6 and -8 further enhances the inflammatory response and induces the production of additional cytokines, thus facilitating the inflammation-cancer cycle (78). TTP can negatively regulate many inflammatory and oncogenic cytokines (3). Therefore, inhibiting inflammation through TTP may help prevent the occurrence and progression of cancers.

NF-κB and STAT3, activators of transcription, collaboratively link inflammation to cancer (79). Compared with normal cells, a sustained inflammatory response has been attributed to negative regulation of the NF-κB-STAT3 pathway in tumors (79). TTP and SOCS3 can affect NF-κB and STAT3, respectively, and inhibit cancerous inflammation in prostate cancer (80). IL-6, which is overabundant in the inflammatory response, has been associated with lethal prostate cancer accompanied by low TTP levels (23). Calcineurin signals stabilize the level of TTP protein by degrading proteasomes, thereby downregulating skin inflammation and inhibiting keratinocyte tumors (81).

P38 MAPK is a key factor that drives TNF-α expression in tumor-associated macrophages at the post-transcriptional level, which is achieved by reducing TTP expression (82). The TNF-α-TTP pathway is controlled by two key factors, p38α MAPK and IL-10 (83). TTP expression is regulated by IL-10 through STAT3, but p38α has two different regulatory functions with TTP in the TME (84), the first being post-transcriptional mRNA decay through TTP phosphorylation, and the second is blocking mRNA translation. Following induction by Toll-like receptor (TLR), DUSP dephosphorylates p38α, which promotes TTP expression and then degrades TNF mRNA transcripts. IL-10 also inhibits TNF expression by restoring TTP expression after TLR induction (82). Collectively, these findings suggest that increasing TTP expression could be an approach for controlling inflammation in the TME.

### TTP Plays an Important Role in Immune Surveillance in the TME

Changes in the status of the immune system *in situ* and activating the metastasis in the TME can both lead to the escape of tumor cells from local and systemic immune control (85). Various immunosuppressive cells and factors in the TME enable cancer cells to evade immune surveillance, except for the cells involved in tumor antigen presentation (86). TME status, including the presence of tumor-infiltrating immune cells, is a decisive factor for cell survival and tumor development (87). The maintenance of immune homoeostasis is mediated by various signaling molecules and involves complex mechanisms (88).

The immunosuppressive protein programmed death ligand 1 (PD-L1) and regulatory T cells (Tregs) play a key role in maintaining peripheral immune tolerance (89). PD-L1 is upregulated in many cancers and helps evade the host immune system (90). Downstream of RAS, MEK promotes kinase MK2-dependent TTP phosphorylation (91). Inactivation of oncogenic RAS-MEK signaling leads to reduction in TTP phosphorylation, stabilizes PD-L1 mRNA, and increases protein abundance. Thus, restoration of TTP expression enhances anti-tumor immunity through the degradation of PD-L1 mRNA (91). PD-L1 plays a crucial role in the infiltration and development of Tregs, a subset of T lymphocytes (92). TTP has a negative effect on Treg infiltration and enhances CD8^+^ T cell cytotoxicity. TTP inhibits Tregs infiltration in gastric cancer by reducing PD-L1 expression, which increases Treg-mediated effector cell cytotoxicity and promotes anti-tumor immunity through CD8+ T cells (92).

**Figure 5:**
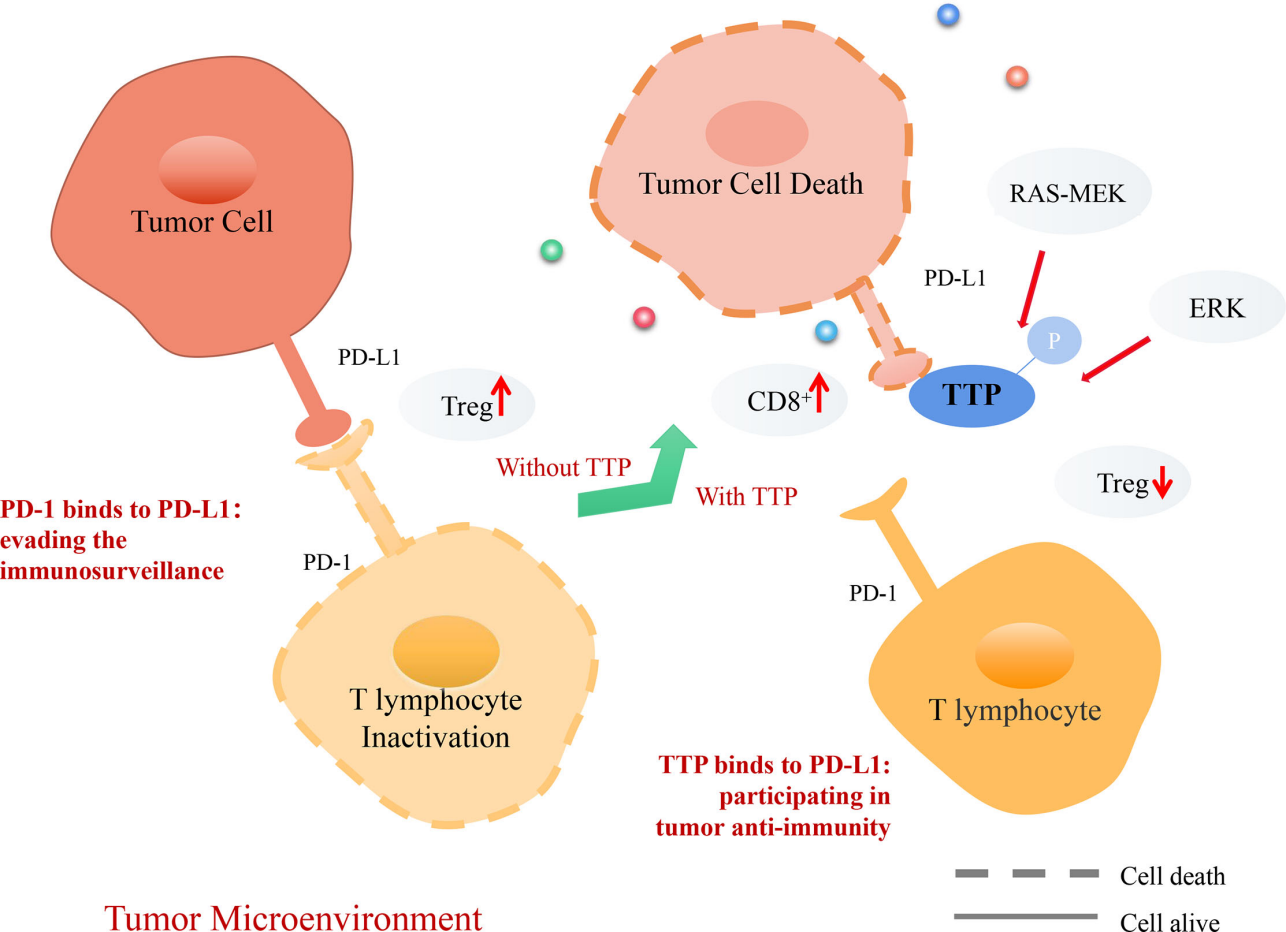
Mechanism of TTP-dependent anti-tumor immunity in the tumor microenvironment (TME). PD-1, a receptor expressed on T lymphocytes, interacts with its ligand PD-L1 on target cells, recognizing healthy cells and preventing induced cell death. However, tumor cells can express PD-L1 and interact with PD-1 on T lymphocytes. T lymphocytes infiltrate Treg cells, evading the tumor immunosurveillance to allow distant metastasis. Consequently, TTP recognizes tumor cells, destabilizes PD-L1 mRNA, and decreases its expression. Thus, T lymphocytes can recognize tumor cells and secrete CD8^+^ cells to promote tumor cell death.

Doxorubicin can reduce PD-L1 expression in cancer cells (93). The underlying molecular mechanism involves anti-tumor immunity, which is achieved by increasing TTP expression and subsequently downregulating PD-L1 expression. Both PD-1 and PD-L1 levels are increased in patients with KRAS-mutant lung cancer (94). In this type of cancer, immune escape is mediated by phosphorylating ERK or by increasing the stability of PD-L1 mRNA (95). The phosphorylation of TTP downstream of ERK causes stabilization of PD-L1 mRNA (93). The relevance of TTP and PD-L1 in the anti-tumor immune response is supported by current evidence (91–94), which demonstrates that TTP could be a novel biomarker in cancer immunotherapy (**Figure 5**).

### TTP Suppresses the Cancer Metabolism

Mammalian cells produce ATP through mitochondrial (oxidative phosphorylation) and non-mitochondrial (glycolysis) metabolism, which provides energy for the cell (96). Cancer cells reprogram nutrient- and oxygen-poor microenvironments to meet their energy and anabolism needs (97). One of the hallmarks of cancer cells is that they enhance the uptake and utilization of glucose, known as the Warburg effect. Cancer cells tend to metabolize glucose through glycolysis, which involves glucose uptake and the subsequent production of glucose-6-phosphate, pyruvate, and lactic acid to support cell proliferation and anabolism (98).

#### Negative Report About TTP Suppressing the Cancer Metabolism

ECM remodeling, during which hyaluronan-mediated motility receptors are closely related to glycolysis, may be considered a basic step in the regulation of extracellular metabolism (99). Hyaluronidase can stimulate the expression of TTP downstream through a receptor tyrosine kinase (RTK)-dependent pathway (100). Then, TTP can mediate the transcription decay of thioredoxin interacting protein (TXNIP) and increase the intake of glucose transporter 1 (GLUT1). At the same time, RTK induces TTP and causes the rapid degradation of TXNIP. This sharp decline in TXNIP induces GLUT1 accumulation on the plasma membrane, thereby inducing the uptake and utilization of glucose (101). TTP-TXNIP-GLUT1 signaling promotes glycolysis by reducing hyaluronan, implying that it is necessary for cancer cells to accelerate migration through ECM decomposition (102).

#### Positive Report About TTP Suppressing the Cancer Metabolism

TTP upregulation decreases the expression of hexokinase 2 (HK2), the first catalytic enzyme of glycolysis. However, when HK2 expression is enhanced in cancer cells, glycolysis and mitochondrial energy production are both reduced. By disrupting the stability of HK2 mRNA, TTP alters the extracellular acidification rate, oxygen consumption rate, and ATP levels of cancer cells, suggesting that TTP is a negative regulator of HK2 expression and glucose metabolism (103). The first rate-limiting enzyme of glycolysis, 6-phosphofructo-2-kinase (PFKFB3), is overexpressed in cancer cells. TTP destabilizes PFKFB mRNA transcripts by binding to the ARE in the 3’ UTR. By downregulating PFKFB3 expression, TTP inhibits energy production and glycolytic flux in cancer cells, increases glutathione expression, and controls the balance between glycolysis and the pentose phosphate pathway at the post-transcriptional level (104).

Notably, TTP promotes glycolysis by increasing the uptake and utilization of glucose in the ECM, whereas in the tricarboxylic acid cycle, TTP inhibits the expression of major enzymes that impede glycolysis in cancer cells. In addition, TTP is also regarded as the promoter of cell metabolism, meaning that TTP provides energy to tumor cells. However, controversy exists regarding the role of TTP in tumor metabolism because some studies claim that TTP may destabilize the mRNA of key enzymes in glycolysis (99, 103). As a result, TTP might reduce the supply of energy to tumor cells. Collectively, these findings suggest that the role of TTP in cancer cell metabolism remains elusive and further research is needed to gain clarity.

## Prospects

The core functions of TTP anti-tumor in the TME include the regulation of inflammation and immunity. As an inflammation-related protein, TTP plays a significant role in cancer onset and progression by modulating the TME, which suggests TTP’s potential mechanism in the transformation from inflammation to tumorigenesis. Moreover, TTP can be regarded as a safeguard against carcinoma due to its role in the TME. Surprisingly, the mechanism is involved in PD-1/PD-L1, the discovery of which won the Nobel Prize in 2018. PD-L1 mRNA degraded by TTP at the post-transcriptional level has the potential in reducing tumor immune evasion. However, a better understanding of the relationship between TTP and TME is required, such as which role TTP plays in regulating metabolism of the TME in the ECM.

In summary, TTP could be a clinical biomarker for cancer diagnosis. Furthermore, upregulating TTP expression may provide a novel approach to improve the prognosis of cancer patients. In 2015, President Obama in U.S.A. has announced the precision medicine project, aiming to provide personalized treatment for individual. With the development of bioinformatics and computer biology, high-throughput sequencing has offered the opportunity to deeply understand many biomarkers including TTP. For example, there have three subtypes of TTP/ZFP36 have been reported, ZFP36*2 (A>G), ZFP36*8 (C>T) and ZFP36*10 (2bp deletion). ZFP36*2 has been proved to be a potential biomarker in Caucasian breast cancer patients while ZFP36*8 has been found high associated with HER2-positive-breast cancer. The availability of accumulated TTP subtype data from patients offers vital and invaluable resources to identify precision treatment for multiple cancer. Further in the gender difference, high TTP level has appeared better overall survival in males than females (105, 106). All above have approved that in the future, a safe and effective way to improve overall survival of tumor patients may be achieved based on the TTP target treatment.

## Author Contributions

JT, DZ and SZ designed and wrote the manuscript. DZ and RY drew the figures. BZ, YT, and LC revised and edited the manuscript. JT, ZZ and TL supervised and checked the review. All authors contributed to the article and approved the submitted version.

## Funding

This work was supported by National Natural Science Foundation of China (82060662), National Innovative projects for University students (g201910555146, g201910555026, 202010601018), Natural Science Foundation of Hunan Province (2020JJ4081), the Foundation for Guangxi Key Laboratory of Diabetic Systems Medicine (20-065-77), Hunan Provincial Key Laboratory of Tumor Microenvironment Responsive Drug Research (2019-56), Hunan Provincial Cooperative Innovation Center for Molecular Target New Drug Study (2016-429), and Shanghai Talent Development Fund (2017116).

## Conflict of Interest

The reviewer ZM declared a shared affiliation with several of the authors, ZZ, LC, JT and the reviewer CKT declared a shared affiliation with several of the authors, DZ, RY, SZ, BZ, YT to the handling editor at time of review.

## Abbreviations

TTP, tristetraprolin; ARE, AU-rich element; TZF, tandem zinc-finger; TNF, tumor necrosis factor; ACC, Adrenocortical carcinoma; BLCA, Bladder urothelial carcinoma; BRCA, Breast invasive carcinoma; CESC, Cervical squamous cell carcinoma; COAD, Colon adenocarcinoma; NF-κB, nuclear factor kappa-B; HDAC, histone deacetylases; Bcl-2, B-cell lymphoma 2; SCCHN, squamous cell carcinoma of the head and neck; HMGB1, enhancement of high mobility group box 1; VEGF, vascular endothelial growth factor; gAcrp, globular adiponectin; HCC, hepatocellular carcinoma; RIP1, receptor-interacting protein kinase 1; FADD, Fas-associating protein with a novel death domain; IAP, inhibitor of apoptosis; GALR2, galanin receptor type 2; GPCR, G-protein-coupled receptor; NaBu, sodium butyrate; TME, tumor microenvironment; STAT3, signal transducer and activator of transcription 3; TLR, Toll-like receptor; PD-L1, programmed death ligand 1; Treg, regulatory T cell; ECM, extracellular matrix; TXNIP, thioredoxin interacting protein; GLUT-1, glucose transporter 1; HK2, of hexokinase 2; PFKFB3, 6-phosphofructo-2-kinase; COX-2, cyclooxygenase-2; RTK, receptor tyrosine kinase.
